# Urinary concentrations of total and free bisphenol A and daily intake estimation in northern Mexican women

**DOI:** 10.1007/s10661-025-14074-7

**Published:** 2025-05-26

**Authors:** Belen Barajas, Ángel Mérida-Ortega, Lizbeth López-Carrillo, Mariano E. Cebrián

**Affiliations:** 1 Departamento de Toxicología, Centro de Investigación y de Estudios Avanzados del Instituto Politécnico Nacional, Ciudad de Mexico, C.P 07360 Mexico; 2 Instituto Nacional de Salud Pública, Col. Santa María Ahuacatitlán, Av. Universidad 655, C.P. 62100 Cuernavaca, Morelos, Mexico

**Keywords:** Free bisphenol A, Total bisphenol A, BPA, Urine, Women, Mexico

## Abstract

Bisphenol A (BPA) is a component of polycarbonates used to manufacture plastic reusable bottles and miscellaneous containers; it is a constituent of epoxy resins used for the internal coating of beverage cans. Humans are mainly exposed by ingesting contaminated food and beverages. Experimental and human studies reported diverse health problems associated with BPA. Our objectives were to measure free (FBPA) and total (TBPA) urinary levels, to estimate daily BPA intake, and to investigate sources of exposure in northern Mexican women. This study is a secondary cross-sectional analysis in controls (*n* = 201) participating in a population-based case–control study. BPA was extracted from urine using the Micro-QuEChERS method and determined by gas chromatography/mass spectrometry. FBPA was detected in 88% of participants (geometric mean: 6.66; range: < LOD—48.83 ng/ml) whereas TBPA was detected in 98.5% (20.13; < LOD—37.15 ng/ml). TBPA estimated intake was 0.199 (0.007–4.26 μg/kg/bw/day), lower than the current American and Canadian tolerable daily intakes (TDI) of 50 and 25, respectively, but higher than the recently proposed TDIs by the European (0.0002 μg/kgbw/day) and German (0.2 μg/kgbw/day) authorities. Women having lower FBPA levels presented lower energy consumption, whereas those with higher TBPA concentrations were younger, had higher BMI, and higher energy intake. TBPA was positively associated with the estimated intake of sugar-sweetened beverages (SSB) but significantly negatively associated with cow’s milk. The urinary concentrations of FBPA and TBPA indicated that women were widely exposed to BPA since their values were in the upper part of the range reported in other regions.

## Introduction

Bisphenol A, known as BPA (4,4′-(propane-2,2-diyl)diphenol), is an organic molecule made from two phenols connected with a methyl group and two functional methyl groups, used as a component of polycarbonate plastics and epoxy resins (Kang et al., 2006). BPA is considered lipophilic due to its low water solubility and high octanol–water partition coefficient (3.32), does not form strong chemical bonds, but only interacts physically with products, so it can easily leach, migrate, and/or evaporate from materials containing it—processes that increase migration are related to plastic age, alkaline conditions, and heating patterns (Talsness et al., 2009). BPA is in contact with beverages and foods, as it is used in the manufacture of polycarbonates intended to contain them, such as in reusable plastic bottles, baby bottles, plates, cups, and containers for storage or cooking. Resins are used for lids and the internal coating of beverage containers, including the internal film coating of can walls (Kang et al., 2006). Epidemiological studies have reported BPA in serum and adipose tissue, and associations with a variety of health problems, including infertility, weight gain, behavioral changes, early-onset puberty, prostate and mammary gland cancers, cardiovascular effects, and diabetes (EFSA, 2023).

The general population is mainly exposed through consumption of contaminated food and beverages, and direct contact with materials containing BPA (Karahalil & Kordbacheh, 2023). In humans, once BPA enters the body orally, it goes through a first-pass metabolism occurring in the intestine wall and liver, whereas hepatic phase II metabolism produces BPA conjugated with glucuronide (BPAG), and to a lesser extent with sulfate. These conjugated derivatives comprise most of the BPA found in blood, are highly soluble in water, and are excreted in urine. The non-conjugated fraction is known as free BPA (BPAF), and the sum of both fractions is known as total BPA (TBPA) (Skledar & Mašič, 2016). BPAF competes more effectively for binding to ERβ but induces ERα- and ERβ-mediated gene expression with similar efficacy, whereas BPAG does not show any in vitro estrogenic activity (Matthews et al., 2001). These findings have suggested that FBPA measurement in blood provides a better approximation to the biologically active dose; however, FBPA has a short life, particularly limiting for blood spot samples, and its low concentrations in most cases are difficult to distinguish from potential background contamination. Therefore, TBPA in urine is favored as an exposure biomarker because it is specific, less invasive, allows for determining several forms of BPA, and analyzes a considerable number of samples in large studies (Ougier et al., 2021).

A recent systematic review and meta-analysis concluded that there is wide geographic variation in TBPA urinary concentrations in women from different countries, whose medians ranged from 0.75 to 3.20 ng/ml (Colorado-Yohar et al., 2021). In Mexico, Indigenous women from Huasteca Potosina showed a mean urinary concentration of TBPA of 0.6 ng/ml (Rodríguez-Báez et al., 2022), whereas an average of 36.3 ng/ml was reported in non-diabetic pregnant women from Mexico City (Martínez-Ibarra et al., 2019). However, information regarding urinary concentrations of FBPA is less abundant, even though information from small studies with the purpose of validating their analytical methods is available. For example, a study in 15 Korean women reported a mean concentration of 0.56 ng/ml and a range of 0.068–1.65 ng/ml (Kim et al., 2003), while a study in 31 German women reported a range of concentrations of < 0.3–2.5 ng/ml (Völkel et al*.,* 2008). In Mexico, a mean urinary concentration of 1.97 ng/ml of FBPA was reported in control women (*n* = 404) participating in a case–control study for breast cancer (López-Carrillo et al., 2021). The European human biomonitoring initiative proposed a guidance value (230 μg/l) for the general adult population (HBM-GVGenPop) of TBPA in urine (Apel et al., 2020; Ougier et al., 2021), considered to provide information on exposure occurring through various routes (Lyu et al., 2023). It is noteworthy that values reported in the literature, some associated with adverse health effects, are below the proposed HBM-GVGenPop, even though this value has not been reaffirmed (EFSA, 2015, 2023). TBPA urinary levels have been considered to provide a reasonable surrogate to estimate BPA daily intake (Lakind & Naiman, 2008) and hence be used for comparison with tolerable daily intake (TDI) values, for example with the recently established TDI of 0.0002 μg/kg bw/day (EFSA, 2023).

On the other hand, variation in urinary BPA concentrations may be due to geographic differences in the consumption of certain foods and beverages, as well as age, gender, and income (Morgan et al., 2018; Nelson et al., 2012). Milk is among the foods consumed daily that, in other studies, represented a high proportion of the BPA intake released by packaging (Fasano et al., 2015; Wan et al., 2023), besides carbonated beverages (Cao et al., 2009) and bottled water (Akhbarizadeh et al., 2020). No specific numerical half-life in food matrices has been universally established for the leached BPA. However, it is considered that BPA does not degrade rapidly in packaged foods but can diminish slowly depending on storage and environmental conditions (Talsness et al., 2009). In Mexico, there is very little information available on the concentration of BPA in foods and beverages, although a range of 0.001–0.021 μg/kg has been reported in dairy products and different canned vegetables (González-Castro et al., 2011). Given the limited information about urinary levels of the different forms of BPA and their association with food and beverage consumption in Mexican populations, the objectives of this study were to quantify FBPA and TBPA in the urine of Mexican women living in northern Mexico, to estimate daily BPA intake, and to investigate possible sources of exposure.

## Materials and methods

### Study design and population

This paper is a secondary cross-sectional analysis of urinary BPA concentrations and their possible sources of exposure in control women participating in a population-based case–control study conducted in five states of northern Mexico from 2007 to 2011. The objective of the original study was to evaluate the association of breast cancer (BC) with exposure to environmental pollutants (López-Carrillo et al., 2010, 2014), in which 1045 patients with histopathologically confirmed BC were identified. Women without cancer (1030), with a minimum age of 18 years, no history of cancer, and at least one year ofresidency in the study area participated as controls. The selection of these women was carried out through the Master Sample Framework used by the Ministry of Health for National Health Surveys, which provides a representative list of households and their geographical location (Tapia-Conyer et al., 1992). The response rate was 99.7%. The Ethics, Research, and Biosafety Committees of the National Institute of Public Health approved the original study, and informed consent was obtained from all participants. From the total sample of 1030 women without cancer (controls), due to economic limitations, this report only includes a subsample of 201 women with available information on urinary concentrations of FBPA and TBPA and not the remaining 829, which we termed not included.

### Determination of bisphenol A

First morning urine was collected in BPA-free sterile polypropylene containers. A 5-ml aliquot was deposited in a cryovial (Simport Scientific, Belolie, QC, Canada) and stored at − 70 °C until analysis. Stock solutions (100 mg/L) were prepared in methanol, and working solutions were prepared daily by appropriate dilution and stored at − 20 °C. Homogenized urine (1.5 mL) was mixed with 25 µl of β-glucuronidase/aryl sulfatase obtained from *Helix Pomatia* (5.5/2.6 U/ml; Roche Diagnostics, Indianapolis, USA), and incubated for 17 h at 37 °C to hydrolyze glucuronide- and sulfate-conjugated BPA (Correia-Sá et al., 2018). The extraction of FBPA and TBPA from urine was performed using the Micro-QuEChERS (Quick, Easy, Cheap, Effective, Rugged, and Safe) method B (6 g magnesium sulfate and 1.5 g anhydrous sodium acetate) (Correia-Sá et al., 2018) with minor adaptations. Analyses were performed using a gas chromatograph Perkin Elmer Clarus 500 coupled to a Clarus 560D mass spectrometer (Connecticut, EUA), operated in the electron impact ionization (EI) mode at 70 eV and controlled by TurboMass System V 6.1. The helium (Infra Ultra High Purity, purity ≥ 99.999%) carrier gas flow was maintained constant (1 mL/min). Injection (1 μL) was carried out in splitless mode. We used an Agilent column HP-5MS (30 m × 0.25 mm I.D., and 0.25 μm film thickness). The GC oven temperature was programmed from an initial temperature of 215 °C, ramped at 5 °C/min up to 245 °C. This program resulted in a run time of 6 min. The retention times for BPA and BPA-d16 were 5.27 and 5.20 min, respectively. Other optimized parameters included a transfer line temperature of 250 °C and an ion source of 250 °C. BPA was quantified in the selected ion recording mode (SIR). The selected ions for BPA were 357 and 372, and for BPA d-16 were 368 and 386. The identification was confirmed by retention times and ion ratios. A seven-point matrix-matched calibration curve (0.5, 1, 3, 5, 10, 50, and 100 ng/ml) was prepared using a donor pool urine fortified after extraction. Concentration points were injected in triplicate to obtain inter- and intra-day variation coefficients of 6.2 and 11.3%, respectively. The analysis was performed in series containing 12 samples; each sample was enriched with 10 ng deuterated BPA-(d16) as an internal standard for quality control purposes; average recoveries were between 87.9 and 97.3%. In addition, from each batch, a randomly selected sample was analyzed in duplicate; variation coefficients ranged from 0.1 to 9.0%. A procedural blank obtained from the mentioned pool was also included. Detection limits (DL) for BPA and BPA d-16 were respectively 0.90 and 0.79 ng/ml. For samples with BPA concentrations below DL, the value corresponding to DL divided by two was assigned (Hornung & Reed, 1990). In this study, 11.9% of samples were below DL. We determined urinary creatinine concentrations by spectrophotometry using a commercial kit according to the manufacturer’s instructions with a detection limit of 1 mg/dl (Randox, Antrim Country, UK).

### Estimation of daily BPA intake

Intake was estimated from TBPA urinary concentrations, as described by LaKind and Naiman (2008), using the volume of urine excreted per day (1200 ml) according to Paquet et al. (2015). 

$$\frac{Total\;BPA\;in\;urine\;(ng/ml)\;\ast\;Amount\;of\;urine\;(ml/day)}{Body\;weight\;(kg)}$$

### Assessment of sociodemographic, reproductive, and dietary characteristics

Women were interviewed face-to-face at home by trained personnel and collected information on their sociodemographic and reproductive characteristics by questionnaire; they were also measured and weighed to calculate their body mass index (BMI). The potential sources of BPA evaluated were food consumption frequency through a previously validated questionnaire (Hernández-Ávila et al*.*, 1998; Galván-Portillo et al., 2011), where participants’ usual diet during the year prior to the interview was investigated. The questionnaire had predetermined portions of 119 foods and 14 dishes, with ten answer choices ranging from “never” to “six or more times a day.” We previously obtained the energy content of each food from the Tables of Nutrient Composition No. 20 of the United States Department of Agriculture (USDA, 2007), and those of the Salvador Zubirán National Institute of Nutrition (1 st edition) (Muñoz de Chávez et al., 1996). For each participant, daily intake of total energy was estimated based on food portion size and its frequency of consumption. For the purposes of this work, those foods with a minimum consumption of 0.10 servings per day were considered for analysis.

### Statistical analysis

The distribution of sociodemographic and reproductive characteristics for both women included and not included in this report was estimated and compared using *Student’s t*, *Mann–Whitney’s U,* or *χ*^2^ statistical tests. Additionally, FBPA and TBPA concentrations were in transformed, and their associations with each food of interest were evaluated using linear regression models. Food consumption was classified into deciles and adjusted by energy (Willett et al. 1997). Variables related to BPA were included in the multivariate models (age, BMI, residence, energy, and water consumption). We performed a sensitivity analysis, where we also included urinary creatinine concentrations as an independent variable. We considered a value *α* = 0.05 and used the statistical package Stata version 13 (Stata Corp, College Station, TX, USA).

## Results

Participant women had an average age of about 54 years, had an average period of residency in the study area of 50.4 ± 13.7 years, and most were postmenopausal (62.2%). Their average BMI was 31, they consumed about 2200 cal per day, and had a median of 6 years of education. Regarding reproductive characteristics, they presented menarche at 13 years on average, a median of 4 for the number of children, and 42 months total lactation duration (Table 1). The sociodemographic characteristics of included and non-included women in this study showed no significant differences, except for their distribution by state of residence (Table 1).

**Table 1:** Comparison of sociodemographic characteristics between women included and non-included in the study*

Characteristics	Included	Non-included	*P*-value
	(*n* = 201)	(*n* = 829)	
Age [years], mean ± SD	53.86 ± 11.83	53.76 ± 12.87	0.925
BMI [kg/m^2^], mean ± SD	31.07 ± 5.70	30.49 ± 6.31	0.241
Age at menarche [years], mean ± SD	13.08 ± 1.55	13.01 ± 1.59	0.574
Parity [no. of children], median (p10, p90)	4.0 (2.0, 10.0)	4.0 (2.0, 9.0)	0.373
Total lactation [months]^1^, median (p10, p90)	42.0 (4.0, 168.0)	36.0 (4.0, 158.0)	0.775
Education [years], median (p10, p90)	6.0 (1.0, 9.0)	6.0 (1.0, 11.0)	0.238
Energy [kcals per day], mean ± SD	2126.56 ± 760.79	2075.79 ± 753.44	0.393
Menopause, %			
Pre-menopause	31.84	34.02	0.558
Post-menopause	68.16	65.98	
Residence, %			
Sonora	18.41	25.88	**0.001**
Nuevo León	25.37	29.63	
Chihuahua	25.87	14.75	
Coahuila-Durango	30.35	29.75	

^1^Excluding nulliparous women. *BMI* body mass index, *SD* standard deviation*Due to economic limitations, from the total sample of control women, we only analyzed for FBPA and TBPA a subsample of 201 women (included) and not the remaining 829 women (non-included)

**Fig. 1:**
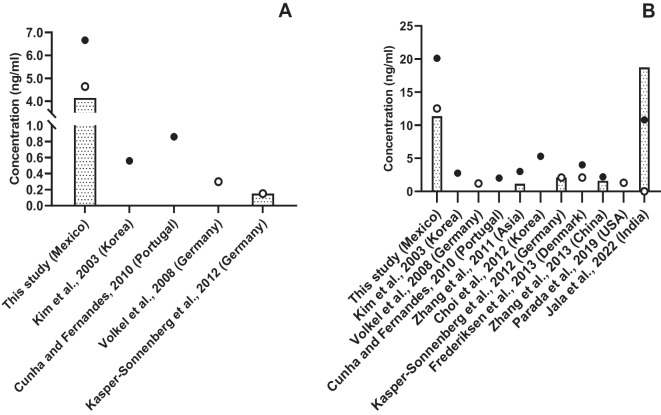
Urinary bisphenol A concentrations in non-pregnant women from different countries. **A** Free BPA. **B** Total BPA. Mean (●), median (○) and geometric mean (**˗**)

Urinary FBPA concentrations were detected in 88.06% of participants, with a mean concentration of 6.67 ng/ml and a range of < LOD to 48.8 ng/ml. TBPA was detected in 98.51% of the women, showing a mean concentration of 20.1 ng/ml and a range of < LOD to 37.1 ng/ml (Table 2). The mean BPAF and TBPA values were several times higher than those reported in other descriptive studies (Fig. 1). The estimation of TBPA intake showed a geometric mean of 0.199 with a range of 0.007 to 4.26 μg/kg/bw/day).

**Table 2:** Free and total urinary BPA concentrations in northern Mexican women (*n* = 201)

	Correction	% < LOD	Mean ± SD	p25	Median	p75	GM
Free	No (ng/ml)	11.9	6.7 ± 7.1	2.3	4.6	8.2	4.1
	Creatinine (μg/g)		18.4 ± 41.7	3.2	7.1	17.8	7.3
Total	No (ng/ml)	1.5	20.1 ± 29.7	6.1	12.5	23.4	12.0
	Creatinine (μg/g)		48.6 ± 124.7	10.1	19.0	41.9	21.3

The comparison of women’s characteristics according to urinary BPA levels showed that those having lower FBPA concentrations presented lower energy consumption, whereas those presenting higher levels lived in the states of Coahuila and Durango. Similarly, participants with higher concentrations of TBPA were younger, had higher BMI, and higher energy intake (Table 3). To discern possible sources of exposure, we evaluated the association of TBPA with the estimated intake of each food/beverage and only observed a non-significant positive association with the consumption of sugar-sweetened beverages (SSB) but significant negative associations with chili and cow’s milk, and a significant negative association between egg consumption and urinary FBPA concentration (Table 4).In the final multivariate models, the negative association between milk consumption and TBPA remained (*β*_95%CI__=_ − 0.07 (− 0.12, − 0.03) (Table 5). In addition, non-significant positive associations were identified between FBPA or TBPA with bottled water consumption (Table 5). No significant changes in the associations tested were observed after adjusting BPA levels for urinary creatinine concentrations (data not shown in Tables).

**Table 3:** Selected population characteristics according to median urinary bisphenol A concentrations

Characteristics	*(n)*	Bisphenol A (ng/ml)			
		Free		Total	
		< 4.64	≥ 4.64	< 12.54	≥ 12.54
Age [years], mean ± SD	(201)	54.5 ± 10.7	53.2 ± 12.9	**55.8 ± 10.7**	**51.9 ± 12.6**
BMI [kg/m^2^], mean ± SD	(201)	30.7 ± 5.6	31.4 ± 5.8	**30.2 ± 5.3**	**32.0 ± 6.0**
Age at menarche [years], mean ± SD	(201)	13.2 ± 1.5	13.0 ± 1.6	13.0 ± 1.4	13.1 ± 1.7
Parity [number of children], median (p10, p90)	(201)	5.0 (1.0, 10.0)	4.0 (2.0, 10.0)	5.0 (1.5, 10.0)	4.0 (2.0, 10.0)
Total lactation [months]^1^, median (p10, p90)	(197)	42.0 (4.0, 169.0)	39.0 (4.0, 162.0)	39.0 (4.0, 180.0)	46.0 (2.0, 142.0)
Energy [kcals per day], mean ± SD	(201)	**1985.1 ± 631.0**	**2266.6 ± 850.6**	**1988.3 ± 674.4**	**2263.4 ± 818.1**
Menopause, %					
Pre-menopause	(64)	30.0	33.7	26.0	37.6
Post-menopause	(137)	70.0	66.3	74.0	62.4
Residence, %					
Sonora	(37)	**22.0**	**14.8**	21.0	15.8
Nuevo León	(51)	**28.0**	**22.8**	32.0	18.8
Chihuahua	(52)	**29.0**	**22.8**	23.0	28.7
Coahuila-Durango	(61)	**21.0**	**39.6**	24.0	36.6

Bold = *p* < 0.05. ^1^Excluding nulliparous women. *BMI* body mass index, *SD* standard deviation

**Table 4:** Median daily food consumption and beta coefficients for selected foods and beverage consumption with bisphenol A urinary concentrations

Foods	Daily consumption portion	Bisphenol A (ng/ml)	
		Free	Total
	Median (p10, p90)	β (95% CI)	
Corn tortillas	9.0 (3.0, 22.5)	0.01 (− 0.04, 0.07)	− 0.01 (− 0.06, 0.04)
Flour tortilla	1.0 (0.0, 5.0)	− 0.01 (− 0.07, 0.05)	0.01 (− 0.05, 0.06)
Corn oil	1.0 (0.8, 2.5)	0.04 (− 0.02, 0.10)	0.06 (0.00, 0.11)
Egg	0.9 (0.1, 2.0)	** − 0.06 (− 0.11, − 0.01)**	− 0.02 (− 0.07, 0.03)
Onion	0.8 (0.1, 1.0)	0.01 (− 0.04, 0.07)	0.04 (− 0.01, 0.09)
Garlic	0.8 (0.1, 1.0)	0.02 (− 0.03, 0.08)	0.04 (− 0.01, 0.09)
Beans	0.4 (0.1, 1.0	− 0.05 (− 0.11, 0.00)	− 0.03 (− 0.09, 0.02)
Coffee	0.4 (0.0, 1.0)	− 0.01 (− 0.07, 0.04)	− 0.02 (− 0.07, 0.03)
Soda	0.2 (0.0, 1.1)	− 0.03 (− 0.08, 0.03)	0.03 (− 0.02, 0.08)
Chili	0.1 (0.1, 1.0)	− 0.03 (− 0.08, 0.03)	** − 0.06 (− 0.11, − 0.01)**
Milk	0.1 (0.1, 1.0)	− 0.03 (− 0.08, 0.02)	** − 0.08 (− 0.13, − 0.03)**
Rice	0.1 (0.1, 0.8)	− 0.00 (− 0.06, 0.05)	− 0.02 (− 0.07, 0.03)
Chicken	0.1 (0.1, 0.4)	− 0.02 (− 0.08, 0.04)	− 0.05 (− 0.10, 0.00)
Broccoli	0.1 (0.0, 0.4)	− 0.02 (− 0.08, 0.03)	− 0.03 (− 0.08, 0.03)
Corn	0.1 (0.1, 0.4)	− 0.02 (− 0.07, 0.04)	− 0.04 (− 0.09, 0.01)
Potato	0.1 (0.1, 0.4)	− 0.00 (− 0.06, 0.05)	− 0.00 (− 0.05, 0.05)
Carrot	0.1 (0.1, 0.4)	− 0.00 (− 0.05, 0.05)	− 0.01 (− 0.06, 0.04)
Lettuce	0.1 (0.0, 0.4)	− 0.02 (− 0.08, 0.03)	− 0.02 (− 0.07, 0.03)
Salad	0.1 (0.1, 0.4)	− 0.01 (− 0.06, 0.04)	− 0.01 (− 0.05, 0.04)
Paste	0.1 (0.0, 0.4)	− 0.04 (− 0.09, 0.02)	− 0.03 (− 0.09, 0.01)

Model adjusted for age, BMI, residence, energy, and drinking water. n = 201. Bold = *p *< 0.05

**Table 5:** Beta coefficients for bisphenol A urinary concentrations and its potential sources of exposure

Exposure sources	Bisphenol A (ng/ml)	
	Free	Total
	*β* (95% CI)	
Chili (daily portion)	− 0.01 (− 0.06, 0.05)	− 0.05 (− 0.11, − 0.00)
Eggs (daily portion)	− 0.05 (− 0.11, 0.00)	0.01 (− 0.04, 0.06)
Milk (daily portion)	− 0.02 (− 0.08, 0.03)	** − 0.07 (− 0.12, − 0.03)**
Water		
Tap	0	0
Bottled	0.05 (− 0.32, 0.42)	0.25 (− 0.08, 0.58)

Bold = *p *< 0.05. Model adjusted for age, BMI, residence, energy, and variables presented in the table. *n *= 201

## Discussion

Our main findings were the elevated urinary concentrations of FBPA and TBPA in a female Mexican sample, which were among the highest reported in the literature. Information on urinary FBPA values in populations is still limited, emphasizing the need to generate further information on the presence and significance of different types of BPA in urine. In relation to urinary concentrations of TBPA, the higher urinary values were observed in India (Jala et al., 2022) and the present study. It is worth mentioning that several studies on TBPA levels were carried out in North American women undergoing assisted reproduction procedures, in which their association with adverse effects on ovarian function was described (Ehrlich et al., 2012a, 2012b; Mok-Lin et al., 2010; Souter et al., 2013; Zhou et al., 2017).

Regarding age and place of residence variables, the highest concentration of urinary TBPA in our study was found in younger women, probably related to higher food consumption resulting in higher energy intake and BMI. It has been suggested that discrepancies in the magnitude of BPA exposure reported in different studies are likely due to diet and other concurrent factors contributing to variability in exposure through different sources (Morgan et al., 2018; Park et al., 2016). In the present study, the geometric mean of daily BPA intake estimated from urinary concentrations (0.199 μg/kg/bw/day) was higher than that reported for 30 countries in a similar study (Huang et al., 2017); however, it was lower than the TDIs of 50 and 25 μg/kg bw/day (U.S. EPA, 2010; Health Canada, 2009) but higher than the recently proposed TDIs of 0.0002 μg/kg bw/day (EFSA, 2023) and 0.2 μg/kg bw/day of the German Federal Institute for Risk Assessment (BfR, 2023); these differences were mainly driven by the choice of relevant effects (Kortenkamp et al., 2024). This suggests the need to evaluate the health risks associated with chronic exposure resulting in the urinary concentrations reported here. In this context, it is necessary to identify the sources of exposure, as they could help to explain large variations in BPA concentrations among different studies.

The determination of BPA has been carried out mainly in foods and beverages, although SSB were the most consumed (Sajiki et al., 2007). In Greece, BPA concentrations were reported to range from 0.4 to 10.2 μg/l, whether contained in glass or PET bottles (Tzatzarakis et al., 2017). In Canada, concentrations of 0.1 to 1.5 μg/l in SSB in various presentations, such as cola, flavored, diet, caffeine-free, or low-sodium, were reported (Cao et al., 2009). Studies in Italy indicated that SSB in different presentations showed BPA concentrations of 0.54 to 4.98 μg/l, with the highest values observed in canned cola drinks (Fasano et al., 2015). Although no information is available about levels in Mexican SSB, it is likely that they contain BPA, as it occurs in other countries, consequently being a source of frequent oral exposure, since our participants reported high daily SSB consumption. This was in accordance with previous Mexican reports, where 80% of women consumed ≥ 1 serving of 250 ml/day, with a median of 489 ml/day of SSB (Martinez et al., 2018). Another study in 394 students (18–32 years old) in Central Mexico reported a weekly consumption of 1572 ml, and only 0.1% of participants mentioned not consuming SSB (Campos-Ramírez et al., 2024). Both studies support the idea that Mexico is among the highest SSB consumers in the world. This suggests the need to study the relationship between BPA concentrations in these beverages, volume consumed, and urinary levels in exposed populations. Another beverage highly consumed is cow’s milk (Wan et al., 2023), in China, several brands were evaluated and TBPA was detected in 10% of the samples showing an average concentration of 0.49 μg/kg (Shao et al., 2007); however, various brands were evaluated in Japan without detecting BPA (Kang & Kondo, 2003; Sajiki et al., 2007). In Mexico, an average milk consumption of 135 ml/day was reported for women over 18 years of age, contributing to ~ 8% of the total fluids ingested (Martinez et al., 2018). Milk is sold mainly in cardboard packages, in plastic bottles and bags. However, little information is available on BPA milk content or leaching from these containers. A possible explanation for the negative association here reported is that calcium and lipid via higher milk intake could impair BPA absorption. Another explanation is related to container characteristics, both cardboard and plastic containers may leach bisphenol A (BPA) into food and beverages, but the likelihood and extent depend on several factors, including the materials used, manufacturing processes, and conditions of use. The pH of food or beverages stored in plastic containers significantly influences BPA leaching. For example, SSBs are notably acidic (pH 2.3 to 3.5) enhancing leaching into the beverage. Conversely, fresh cow’s milk typically has a stable, slightly acidic pH (6.4 to 6.8) not significantly promoting BPA leaching from plastic containers. Furthermore, heating food can increase the rate of BPA migration into the food. In a recent review, exposure to BPA through bottled water consumption was estimated at 0.3 μg/kg bw/day in the worst-case scenario, suggesting that container types play an important role in the magnitude of exposure (Akhbarizadeh et al., 2020); however, in Mexico, there was no information available on BPA concentrations in bottled water.

Among the limitations of this study is the collection of a single urine sample, which may not represent the variability of the values throughout the day and could be attributed to episodic exposure, though the variability was suggested to be comparable to that shown by the average concentrations of 24 h (Christensen et al., 2012; Ougier et al., 2021). In addition, the biological half-life of FBPA and CBPA in humans after oral intake is short (~ 6 h) (Skledar & Mašič, 2016); however, ongoing exposure through food and the environment can lead to a constant low-level presence. Regarding FBPA, their urinary levels are low in the general population and may be difficult to distinguish from the background levels. Another limitation is the absence of available information on the BPA content of foods and beverages considered as possible sources of exposure and leaving out inhalation and dermal exposure. There were small differences in the percentage of women coming from the states of Sonora and Chihuahua; however, selected characteristics of women included and non-included in the study did not differ significantly, so we consider low the probability of having a biased sample. In addition, we selected the original sample with a probabilistic approach, increasing the probability that our results might be extrapolated to women residing in the study region. Our sample size may not allow us to adequately estimate BPA intake and/or identify other potential dietary-BPA associations, as it has been suggested that larger samples are required (Huang et al., 2017.

## Conclusion

To our knowledge, this study is the first to show urinary concentrations of both FBPA and TBPA in Mexican women residing in northern Mexico, indicating that they are widely exposed to BPA, since their levels were in the upper part of the range reported in the literature and are also within the range associated with various adverse effects described. Our calculated TDI was higher than that reported previously for 30 countries, but lower than the values proposed in the USA and Canada) and higher than those proposed recently by EFSA and the BfR. Taken together, our results and those from the literature give evidence of the presence of BPA in women and its presence in products regularly marketed. In Mexico, there is a record of a legislative petition initiative to regulate BPA that has not yet resulted in public policy change (Mandel et al., 2019). Further studies in Mexico are needed regarding BPA levels in packaged and canned foods and beverages. The frequency of their consumption is also an important element for the study of the relationship between exposure and its possible health effects. An agreement in the process of establishing TDIs would be useful for developing countries attempting to advance regulations.

## Data Availability

No datasets were generated or analysed during the current study.
